# A Comparison and Calibration of a Wrist-Worn Blood Pressure Monitor for Patient Management: Assessing the Reliability of Innovative Blood Pressure Devices

**DOI:** 10.2196/jmir.8009

**Published:** 2018-04-25

**Authors:** Sarah Melville, Robert Teskey, Shona Philip, Jeremy A Simpson, Sohrab Lutchmedial, Keith R Brunt

**Affiliations:** ^1^ Department of Pharmacology Dalhousie Medicine New Brunswick Saint John, NB Canada; ^2^ Cardiovascular Research New Brunswick New Brunswick Heart Centre Saint John, NB Canada; ^3^ Department of Cardiology Saint John Regional Hospital Horizon Health Network Saint John, NB Canada; ^4^ Department of Human Health Nutritional Sciences University of Guelph Guelph, ON Canada

**Keywords:** patient self-management, diastolic hypertension, telemonitoring, vital signs, smartphone applications

## Abstract

**Background:**

Clinical guidelines recommend monitoring of blood pressure at home using an automatic blood pressure device for the management of hypertension. Devices are not often calibrated against direct blood pressure measures, leaving health care providers and patients with less reliable information than is possible with current technology. Rigorous assessments of medical devices are necessary for establishing clinical utility.

**Objective:**

The purpose of our study was 2-fold: (1) to assess the validity and perform iterative calibration of indirect blood pressure measurements by a noninvasive wrist cuff blood pressure device in direct comparison with simultaneously recorded peripheral and central intra-arterial blood pressure measurements and (2) to assess the validity of the measurements thereafter of the noninvasive wrist cuff blood pressure device in comparison with measurements by a noninvasive upper arm blood pressure device to the Canadian hypertension guidelines.

**Methods:**

The cloud-based blood pressure algorithms for an oscillometric wrist cuff device were iteratively calibrated to direct pressure measures in 20 consented patient participants. We then assessed measurement validity of the device, using Bland-Altman analysis during routine cardiovascular catheterization.

**Results:**

The precalibrated absolute mean difference between direct intra-arterial to wrist cuff pressure measurements were 10.8 (SD 9.7) for systolic and 16.1 (SD 6.3) for diastolic. The postcalibrated absolute mean difference was 7.2 (SD 5.1) for systolic and 4.3 (SD 3.3) for diastolic pressures. This is an improvement in accuracy of 33% systolic and 73% diastolic with a 48% reduction in the variability for both measures. Furthermore, the wrist cuff device demonstrated similar sensitivity in measuring high blood pressure compared with the direct intra-arterial method. The device, when calibrated to direct aortic pressures, demonstrated the potential to reduce a treatment gap in high blood pressure measurements.

**Conclusions:**

The systolic pressure measurements of the wrist cuff have been iteratively calibrated using gold standard central (ascending aortic) pressure. This improves the accuracy of the indirect measures and potentially reduces the treatment gap. Devices that undergo auscultatory (indirect) calibration for licensing can be greatly improved by additional iterative calibration via intra-arterial (direct) measures of blood pressure. Further clinical trials with repeated use of the device over time are needed to assess the reliability of the device in accordance with current and evolving guidelines for informed decision making in the management of hypertension.

**Trial Registration:**

ClinicalTrials.gov NCT03015363; https://clinicaltrials.gov/ct2/show/NCT03015363 (Archived by WebCite at http://www.webcitation.org/6xPZgseYS)

## Introduction

Hypertension is a global health problem affecting over a billion people [1]. If uncontrolled, it is a major risk factor for stroke, myocardial infarction, and kidney failure; it remains the leading cause of cardiovascular morbidity and mortality [2,3]. Diagnosis and management of hypertension relies intensively on the indirect measurement of blood pressure in outpatient settings.

Manual auscultatory and automated oscillometry (ie, clinical and patient blood pressure monitoring methods) are common means for diagnosing hypertension and guiding appropriate medical management. Current guidelines (Canadian Hypertension Education Program, CHEP; American Society of Hypertension; International Society of Hypertension; European Society of Hypertension, ESH) recommend confirmation of hypertension (ie, blood pressure ≥135/85) with ambulatory or home blood pressure monitoring [4-6] . In recent years, there has been a dramatic growth in automated devices and increased use of mobile health apps [7]. However, there is limited information about their accuracy or precision, which creates a risk for inappropriate therapy or a treatment gap [8,9]. Clinically, the term *treatment gap* refers to hypertensive patients who are left untreated because of underestimated noninvasive blood pressure readings, which could be a health risk for the patient.

Automated clinical oscillometric or consumer-level devices have been generally compared with manual auscultatory measurements [10-12], while studies using invasive blood pressure measurements for validation or calibration are increasing [13-15]. Regulatory agencies (eg, Health Canada; Food and Drug Administration, FDA) license devices without mandatory independent third-party, peer-reviewed assessment of the validity of measurements or calibration standards. The principal directive of regulatory agencies for these sphygmomanometer devices (Health Canada and FDA Class II) is to ensure physical safety and personal data security [16,17] rather than guarantee accuracy and precision for clinical diagnostic purposes. The minimal requirements prescribed by Health Canada are not the minimal requirements of a clinician.

Increasingly, automated devices are being assessed against direct intra-arterial standards for clinical certainty, yet, most still do not report the uncertainty of the single blood pressure measurement. Comparing direct and indirect blood pressure measures simultaneously ensures that intraphysiological variability can be accounted for in the measures. The difference between 2 methods can then be validated with multiple measures within a patient population. There is a growing need for more accurate devices to measure blood pressure [18] to better diagnose and manage hypertension according to clinical guidelines. This could be accomplished by calibration to simultaneous direct measures in addition to auscultatory calibration using indirect measurements to reduce the treatment gap in hypertension. Generally, consumer devices, clinical devices, and the true invasive blood pressure measures in healthy and hypertensive patients should be in agreement with each other. Our objective was to assess the validity of indirect measures of blood pressure by a wrist-worn blood pressure device in direct comparison with simultaneously recorded gold standard intra-arterial blood pressure measures for the purpose of iterative device calibration.

## Methods

### Recruitment and Screening

**Figure 1:**
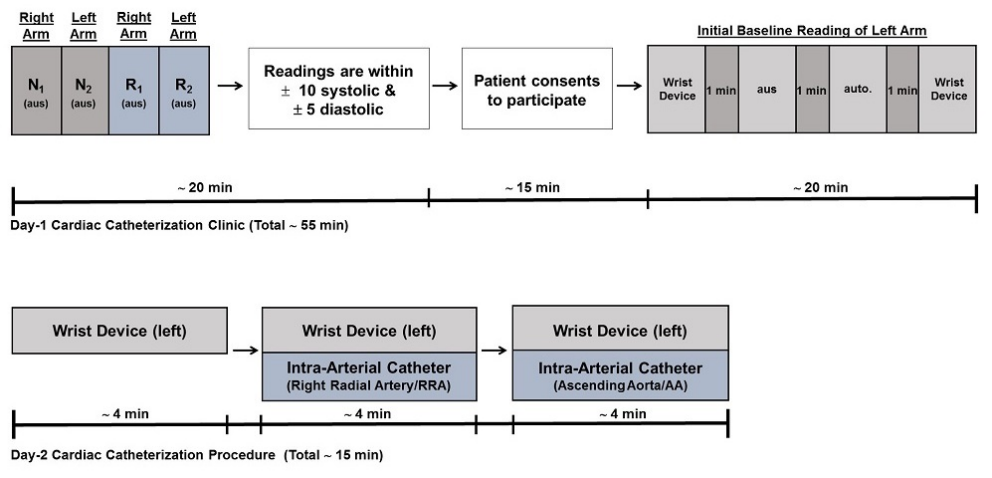
Procedural overview: timelines for patient recruitment and data collection. Day 1-Timeline intake of patients for inclusion screening followed by consent and initial data collection. N and R are 2 trained investigators measuring blood pressure twice using the auscultatory method (aus); initial baseline readings used an automatic upper arm cuff (auto.). Day 2-Timeline for simultaneous invasive and noninvasive blood pressure measurements.

The clinical protocol was approved by the Horizon Health Network Research Ethics Board, and the study is registered with the National Institute of Health Clinical Trials Registry database (NCT 03015363). The patient participant inclusion/exclusion criteria were: (1) a referral from the patient’s attending cardiologist to undergo a first-time nonemergent diagnostic cardiac catheterization procedure for clinically valid indications; (2) participants aged ≥19 years; (3) wrist circumference should be in the range of 13.5 to 23 cm; (3) participant should be willing to voluntarily sign the study-specific informed consent form; and (4) participant should have no previous percutaneous coronary intervention, coronary artery bypass graft, abdominal aortic aneurysm, peripheral vascular disease, aortic stenosis, arrhythmia, tremors (before or during procedure), or carotid bruits. In accordance with the Association for the Advancement of Medical Instrumentation (AAMI) standards for clinical investigation using reference-based invasive blood pressure monitoring [19], we undertook a 2-day protocol (Figure 1).

### Blood Pressure Measurements

Logistically, the wrist cuff cannot be applied to the same arm that is being cannulated for radial pressure. Thus, patients were screened for bilateral upper arm auscultatory blood pressure equality (day 1; within ±10 mm Hg systolic and ±5 mm Hg diastolic pressure; see Multimedia Appendix 1) by 2 separate blinded readings performed using an upper arm aneroid sphygmomanometer (Welch Allyn Canada Ltd, Mississauga, ON) by 2 health professionals (Figure 1). This ensured that all patients did not have undiagnosed peripheral vascular disease to cause arm inequality in blood pressure. All eligible participating patients voluntarily provided documented informed consent. After obtaining the consents, 4 blood pressure readings were taken approximately 1 min apart to establish a baseline (Figure 1); 3 different devices were used for general observational comparison, according to standard procedures or manufacturers’ instructions: (1) an oscillometric wrist cuff device (Cloud Diagnostics Inc, PULSEWAVE Health Monitor, Kitchener ON), (2) an upper arm oscillometric device (Welch Allyn Canada Ltd, Mississauga, ON), (3) and an upper arm aneroid sphygmomanometer (Welch Allyn Canada Ltd, Mississauga, ON). The pulse pressure waveform recorded by the wrist cuff device was digitized at 100 Hz and stored securely on a cloud-based server for subsequent analysis.

On the day of the cardiac catheterization procedure (Day 2), patients were given 1mg of midazolam and 50 µg of fentanyl for presedation ; blood pressure readings were taken as indicated in Figure 1 with the patient in the supine position. A total of 10 readings were taken with 2 measurements for each method (Figure 1); the first set of 2 wrist cuff measures were taken for patient conditioning and were not subsequently used for comparison in this study. The second set of 2 wrist cuff measures were taken while simultaneously recording 2 intra-arterial pressures at the right radial artery. The third set of 2 wrist cuff measures were taken while simultaneously recording 2 intra-arterial pressures at the ascending aorta. Each measurement was treated independently throughout this study (N=160 intra-arterial measurements in total for recalibration; 20 participants). Digital records of previously monitored data in a clinical setting using indirect, double-observer auscultatory measurements were secured for the purpose of clinical comparative analysis (N=375 measurements; 97 participants).

Intra-arterial pressure was measured with a fluid-filled 5 or 6 French gauge catheter (Cordis AVANTI+) attached to a pressure transducer (NAMIC, Navilyst Medical, Marlborough, MA, USA), which is consistent with the recent recommendation by the ARTERY Society task force [20]. The pressure transducer was zeroed before the start of the catheterization procedure. Intra-arterial pressure was recorded with hemodynamic software (MAC-LAB, General Electric Company). To avoid verapamil or heparin-induced changes in vascular tone, both drugs were administered as per standard of care procedure after pressure readings were complete.

Calculated device values of systolic and diastolic pressures were initially derived from the Cloud Diagnostics Inc application reports (original auscultatory calibration). The intra-arterial dataset was split into a training set and a testing set using the jackknife technique, as originally described [21]. The jackknife technique is a power data analysis tool suitable for small original data samples. In a dataset of N readings, the jackknife iterative processing can be described as the systematic resampling of a single reading from the entire dataset to be used as the testing set and the rest N−1 readings are used as the training set. This is repeated N times and during the ith iteration, the ith reading is chosen as the testing set and the rest N-1 readings are chosen as the training set. During each iteration the training set is used to obtain calibration coefficients, which are then used on the testing set. After making algorithm adjustment, new systolic and diastolic pressures were obtained directly from the engineers at Cloud Diagnostics Inc.

### Statistical Analysis and Data Reporting

Data analysis was performed via Bland-Altman (Tukey mean difference) plot analysis [22,23] to assess the accuracy of the wrist cuff relative to intra-arterial measures. Systolic and diastolic validity was set a priori as any 2 measures being within 10 and 5 mm Hg, respectively (based on a normally distributed relevant clinical population) [24,25] (See Multimedia Appendix 2). The absolute mean difference and SD of all Bland-Altman plots are reported as the bias [20,26,27] (Also, see Multimedia Appendix 3). A one-tailed Fisher exact test was used to determine the sensitivity of the device in assessing measures of hypertension versus normotension.

## Results

### Blood Pressure Device Calibration

**Figure 2:**
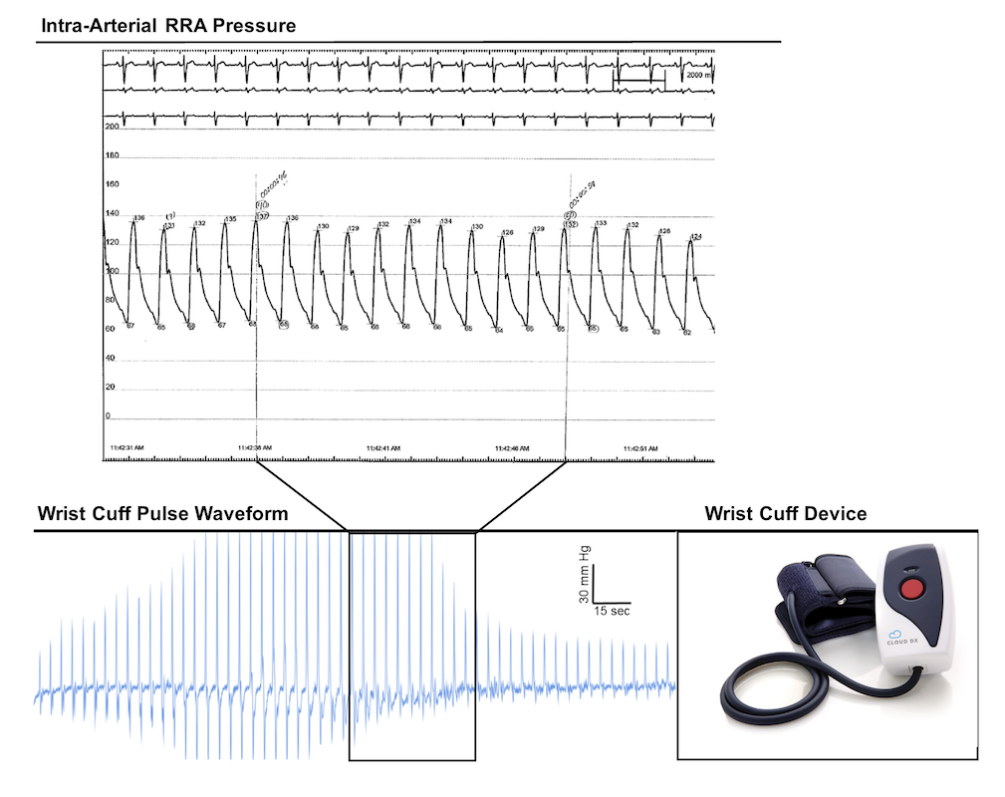
Physiological tracings. Representative hemodynamic tracing from the intra-arterial pressure catheter with electrocardiogram (ECG). Representative pulse waveform report from the wrist-cuff blood pressure device.

Here, we used intra-arterial blood pressure measurements in comparison with the noninvasive wrist cuff method (Figure 2).

A total of 74 potentially eligible patients were screened, of which 37 consented, 3 withdrew, and 14 failed to meet the criteria during the (day 2) procedure. A summary of the patients’ characteristics that completed all aspects of this study is presented in Table 1.

The mean direct right radial arterial systolic pressure was 145.7 (SD 20.2) mm Hg, and the mean direct ascending aorta systolic pressure was 133.4 (SD 22.0) mm Hg. This illustrates the real physiological difference of 14.4 (SD 10.3), with *P*<.001, as a result of pressure augmentation [28]. There was no physiological difference in direct right radial arterial diastolic pressure and direct ascending aorta diastolic pressure (66.2 [SD 9.1] mm Hg vs 67.4 [SD 8.7] mm Hg), respectively; absolute mean difference of 5.1 (SD 3.6), *P*=.23).

**Figure 3:**
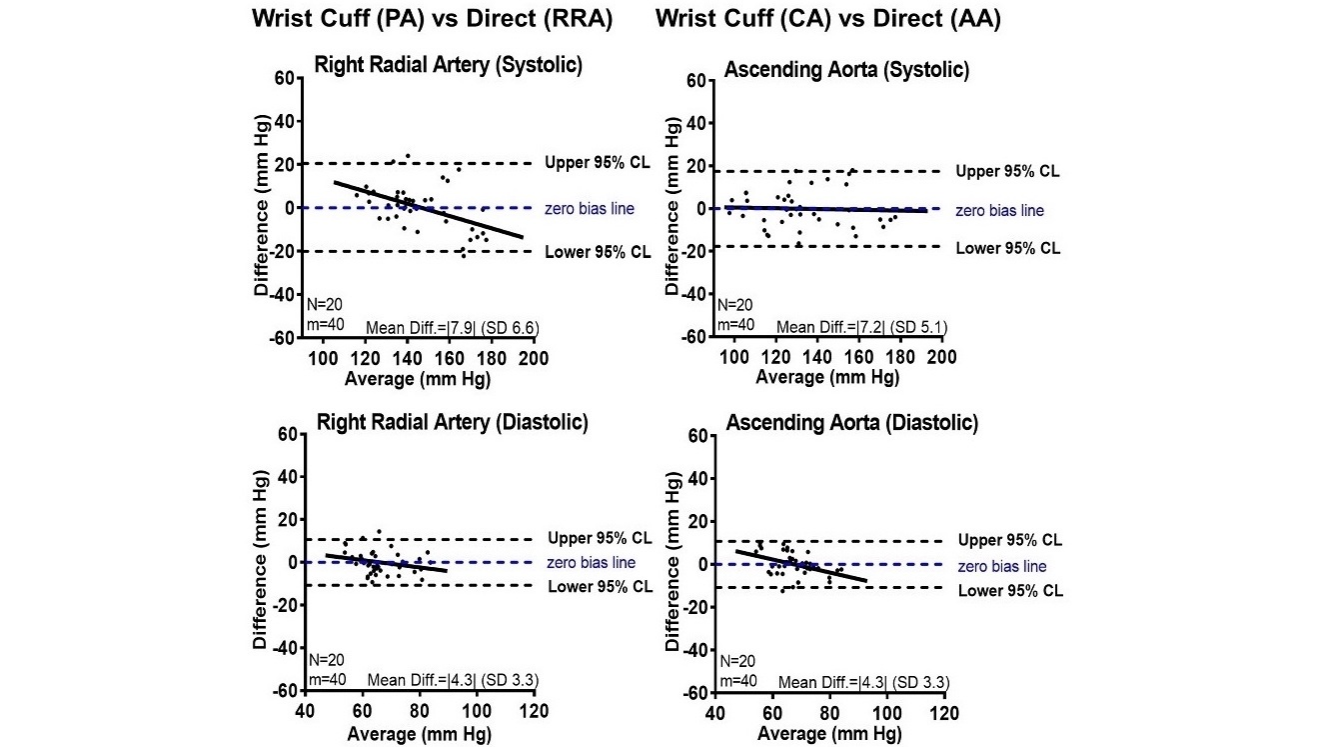
Direct intra-arterial blood pressure agreement with indirect wrist cuff measures. Bland-Altman plot analyses of pressure measurement agreement with: systolic and diastolic blood pressure wrist cuff measurements after peripheral algorithm (PA) adjustment and direct right radial artery (RRA) blood pressure measurements (N=20; mean=80), and systolic and diastolic blood pressure wrist cuff measurements after central algorithm (CA) adjustment and direct ascending aorta (AA) blood pressure measurements (N=20; mean=80).

Initially, the absolute mean difference of the wrist cuff compared with direct systolic measures using the original auscultatory calibration settings was 10.8 (SD 9.7), with *P*<.001, while the absolute mean difference of the diastolic measures was 16.1 (SD 6.3), with *P*<.001 (See Multimedia Appendix 4). Next, we adjusted the algorithm using the intra-arterial blood pressure datasets. First, we applied an iterative calibration of the wrist cuff to radial artery pressures, and the absolute mean difference of the systolic and diastolic measures was 7.9 (SD 6.6), with *P*=.87 and 4.3 (SD 3.3), with *P*>.99, respectively (Figure 3). However, we noted a negative slope trend line that may suggest an attenuated pressure when intraradial systolic pressure is greater than 150 mm Hg. Then, we calibrated the wrist cuff to ascending aortic pressures, and the absolute mean difference of the systolic and diastolic measures was 7.2 (SD 5.1), with *P*=.97 and 4.3 (SD 3.3), with *P*=.98, respectively (Figure 2), with a near 0 systolic pressure trend.

To further assess the value of the central algorithm independently of our calibration dataset, we sourced an arms-length dataset of double-observer auscultatory blood pressure measures from Cloud Diagnostics Inc (375 measurements; 97 participants). The average absolute mean difference of the initial algorithm markedly improved with the central pressure calibrated algorithm in both the systolic and diastolic pressures (7.5 [SD 7.3] vs 6.1 [SD 4.7] and 18.0 [SD 7.6] vs 9.8 [SD 6.0], respectively; see Multimedia Appendix 5). This is an improvement in accuracy of 20% with 38% less variability for systolic measures and 46% more accuracy with 19% less variability for diastolic measures using an independent dataset. Therefore, central pressure calibration improved the accuracy and reliability of the wrist-cuff device comparisons to direct pressures and upper arm cuff measures, which is relevant to clinical practice guidelines.

### Blood Pressure Variability Assessment

**Figure 4:**
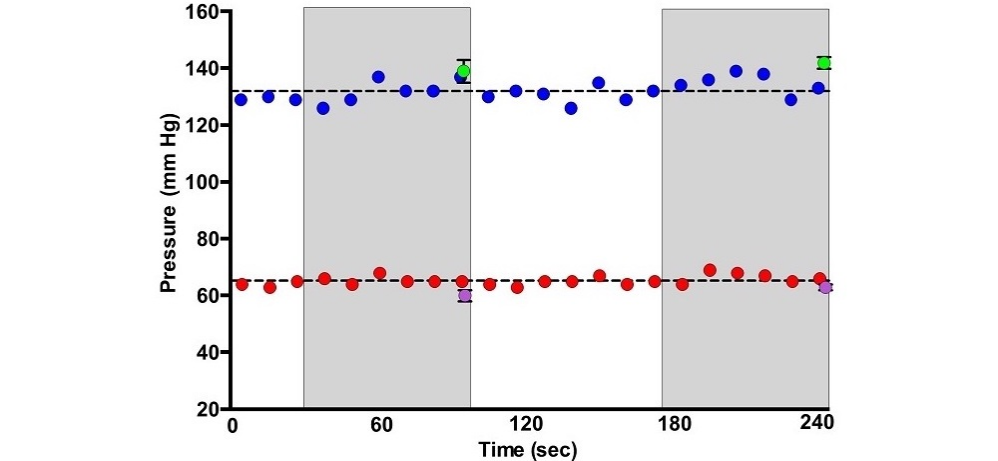
Simultaneous recordings of direct and indirect blood pressures. Representative illustration of instantaneous intra-arterial blood pressures every 10 s over 4 min (blue=systolic; red=diastolic) along with 2 simultaneous readings of wrist cuff blood pressure (green=systolic; purple=diastolic) with SD. Wrist cuff blood pressure is acquired over approximately 1 min (gray boxes).

To demonstrate measurement variability, a representative illustration of instantaneous intra-arterial pressures that were recorded every 10 s over a period of 4 min is shown in Figure 4. Measurements varied by approximately 20 and 10 mm Hg for systolic and diastolic pressures, respectively.

**Figure 5:**
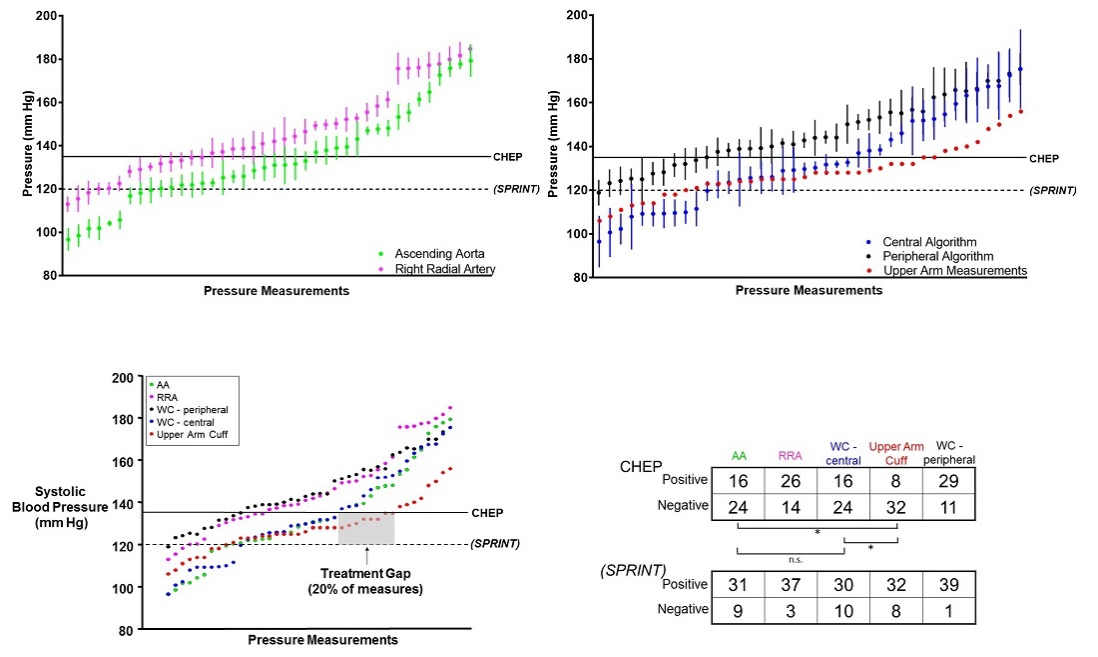
Relevant threshold comparison of peripheral and central pressures, directly and indirectly. Direct intra-arterial systolic pressure measurements from the right radial artery (magenta) and ascending aorta (green). Indirect wrist cuff systolic pressure measurements using the peripheral algorithm (black) and central algorithm (blue); upper arm measurements included with no possibility of a measure of uncertainty. All course of mean systolic measures (without SD) with guideline and trial target thresholds. Note: gray box shows upper arm measures at risk of treatment gap based on Canadian Hypertension Education Program (CHEP) and Systolic Blood Pressure Intervention Trial (SPRINT) thresholds.

Subsequently, all invasive measures having SD 1 were plotted (Figure 5). Direct pressure analysis (both radial and aortic) illustrates pressure augmentation of peripheral blood pressure (Figure 5). Several measures have an SD that crosses the clinical threshold for diagnosing hypertension based on the CHEP guidelines [4] or the Systolic Blood Pressure Intervention Trial (SPRINT) [29]. Similar results were obtained by plotting the indirect measures (Figure 5). Taken together, in the absence of intraphysiological variability reporting (Figure 5), the wrist cuff produces diagnostically similar data to direct arterial pressure with a lower treatment gap risk than a noncalibrated indirect measure, such as the upper arm measures used in this study. Approximately 75% (15/20) of the patients in this study were already diagnosed with hypertension. According to direct intra-aortic and indirect wrist cuff measures, approximately 40% (Figure 5) were above the blood pressure target provided by the CHEP guidelines. When measures were plotted as being either hypertension positive or negative, the centrally-calibrated wrist cuff measures were on par with direct aortic measures using either CHEP- [4] or SPRINT-based [29] thresholds (Figure 5), whereas there was a significant difference between direct aortic pressure measures and calibrated wrist cuff measures compared with the upper arm cuff (*P*=.04; see Figure 5).

**Table 1:** Patient participant characteristics.

Characteristics		Patient participants (N=20)
Sex (male/female)		15/5
Age in years, mean (SD); range		62.0 (SD 9.0); 43-77
BMI^a^, mean (SD); range		30.6 (SD 5.7); 21-45.4
**Central aortic pressure (mm Hg)**		
	Systolic, mean (SD); range	133.4 (SD 22.0); 96.7-179.3
	Diastolic, mean (SD); range	67.4 (SD 8.7); 50.6-85.1
**Peripheral arterial pressure (mm Hg)**		
	Systolic, mean (SD); range	145.7 (SD 20.2); 113.0-184.8
	Diastolic, mean (SD); range	66.2 (SD 9.1); 49.4-84.8
Wrist circumference in cm (left), range		15.5-21.5
Smoking, % (Y/N)		15 (3/17)
Diabetes, % (Y/N)		35 (7/13)
Statin, % (Y/N)		70 (14/6)
Hypertension, % (Y/N)		75 (15/5)

^a^BMI: body mass index.

## Discussion

### Principal Findings

In this study, we assessed the validity of indirect measures of a novel wrist cuff blood pressure device in direct comparison with simultaneously recorded gold standard intra-arterial pressures. This study shows that a calibrated wrist cuff blood pressure device is in agreement with the precision and accuracy of intra-arterial pressure measurements in an in-patient setting. To our knowledge, this study is the first to test and incorporate iterative calibration of a commercially available blood pressure device and also the first to show measurement uncertainty in the device output. This study shows that an automated upper arm device when compared with other indirect methods was less accurate than the gold standard calibration used here; this could reduce the risk of a treatment gap. A calibrated wrist cuff device, if used properly, is capable of producing measures that are clinically accurate for the management of systolic hypertension in accordance with the guidelines for home blood pressure monitoring. In our center, we identified a clinical need to use wrist cuff devices for patients in intensive care (eg, minimize sleep disturbance) or with physical limitations to upper arm cuff use (eg, frailty, obesity). To achieve broad clinical reliability, additional use and monitoring of the device, such as in clinical trials, is warranted.

### Clinical Relevance

This wrist cuff device offers several advantages in comparison with many upper arm devices for the measurement and medical management of hypertension. A common complaint among patients and front-line health care providers, especially when performing repetitive blood pressure monitoring, is that they experience pain with upper arm compression [30]. Frail patients are more likely to become physically intolerant to multiple daily measures with an arm cuff. Furthermore, these devices, when activated in hospitals at night, disrupt essential circadian sleep quality [31]. Obese, senior, and frail patients most often experience cuff malposition issues, either from obese, conical upper arms or from mobility or dexterity issues for self-fastening and positioning [30]. Patients with conical or obese arms and the frail elderly are key target demographics in need of hypertension identification and management.

Individual blood pressure measures from this wrist cuff device report the degree of uncertainty in each measure. While several mean blood pressure measurements were below the threshold for hypertension based on the CHEP guidelines [4] or the SPRINT trial target [29], they were within 1 SD of the threshold. This poses the question as to whether these patients would benefit from more aggressive therapeutic intervention. Future work should assess the frequency by which this occurs in an outpatient setting using a longitudinal study.

### Calibration Issues With Indirect Oscillometric Devices: Peer Review, Interdependence on Therapeutics, and the Level of Uncertainty

The variability between radial and aortic pressures is a function of differences in compliance, vasoactivity, and pressure augmentation, which become more variable with aging and disease [25,28,32]. Thus, noninvasive blood pressure can be calibrated to either radial artery or aortic pressure. Specifically, a universal protocol that recommends intra-arterial pressure as a reference standard to validate noninvasive blood pressure is currently being developed via collaboration of the AAMI and the ESH [20]. While this wrist cuff blood pressure device was calibrated to aortic pressures [20,33-37] in this study, we also presented wrist cuff blood pressure data calibrated to either radial artery or aortic pressures for scientific interest. This natural intraphysiological variability from the mean (Figure 4, dashed line) is derived from variability of neuro-endocrine stimulation, arterial tone, heart rhythm, cardiac output, and respiration. Thus, the correct and accurate way to calculate blood pressure should include intrinsic measures of variance [38] (measure of uncertainty or SD). This was achieved for the first time with a blood pressure device that reported an SD for each measure.

Blood pressure devices are almost exclusively calibrated to manual auscultatory measurements [10-12,17]. In addition to the calibration inconsistency among blood pressure devices as previously described [18], unreliable measurements among automated blood pressure devices are common [39,40]. Most protocols for device comparison are focused on variability within a population rather than variability of blood pressure measures within an individual. Most automated devices currently do not report the uncertainty of the blood pressure measure. We also observed that upper arm measures were not in agreement with aortic pressures and could produce a treatment gap of approximately 20%. The need to align blood pressure measures with the medical management of hypertension is of paramount importance [41]. Additionally, many (medical or life) insurers have a vested financial interest in the reliability of blood pressure as an index of health in determining premiums and eligibility. Fundamentally, the management of hypertension and blood pressure devices are interdependent, yet a barrier to achieving optimal disease management is, at least in part, related to the current lack of information about their precision, accuracy, and level of uncertainty. Indeed, systematic review of noninvasive blood pressure devices reportedly meeting the engineering standards of either the AAMI, ESH, or British Hypertension Society protocols [19,42,43] are inconsistently adhered to and are not always in agreement [18]. Often, incongruent variables are reported in the Bland-Altman analyses that invariably lead to a mean difference of 0 with increasing n-values, which is statistically unacceptable [27], whereas clauses in the protocol can allow for removal of potentially relevant measures (ie, 12/8 rule) [44]. Furthermore, engineering and clinical standards are not comparable—the former is concerned with device reproducibility, while the latter is concerned with interpatient and intrapatient variability. Regulatory agencies (eg, Health Canada, FDA) that license these devices are responsible only for aspects relating to product safety and the comparability to other market devices and not for determining the validity of the measurements, [16,17]. More efforts are required to advance device quality and functionality using a patient-centered approach to accommodate the interdependencies between blood pressure devices and (clinical trial-approved) therapeutics for the management of hypertension [8,9]. A universal protocol that is clinically practical and can consistently determine device validity, including when challenged by a direct pressure analysis, is currently being developed and estimated to be released in 2018 [20].

### Study Limitations and the Impact of Precise and Accurate Blood Pressure Measures on Current Clinical Guidelines

Further calibration studies using this device should include special populations such as pediatric, obese, or frail patients, or those with an underlying arrhythmia or peripheral vascular disease. Also, patients with peripheral movement artifacts (eg, tremors) represent an incremental challenge to wrist cuff devices that would be less pronounced for upper arm cuff devices. Finally, more work is required to determine whether diastolic pressure and diagnoses using indirect diastolic pressure measures are required to be accurate to direct central pressures.

To diagnose hypertension according to guidelines, we must be confident with device measures [45,46]. It is encouraging that large clinical trials have demonstrated improved outcomes in populations achieving even 1 mm Hg reductions in blood pressure, yet devices have no provision for this single digit resolution for an individual patient. Guidelines tend to utilize 5 mm Hg increments and we are not aware of any device that has this resolution without applying variability filtering [44]. Indeed, intrapatient variability of blood pressure within a short period of time can vary by increments of >10 mm Hg, notwithstanding intraday variability of 25 mm Hg or more. This not only necessitates serial averaging of blood pressure measures but also compounds the standard error in the mean of measures. A greater awareness of uncertainty could help to establish criteria of acceptability in a measure. Now, the question, given the recent results of the SPRINT trial [29], whether the lowering of the blood pressure target was achieved by having patients at 120 mm Hg or the consequence of greater certainty in patient catchment below the existing 135 mm Hg threshold will have to be faced. Future clinical trials should report the devices used for blood pressure analysis and their level of uncertainty. This will provide an opportunity to factor in-device uncertainty as guidelines are further refined.

### Conclusions

The wrist cuff calibrated here to the gold standard—the central (ascending aortic) pressure—is an accurate device that can be used in accordance with guidelines for informed decision making in the management of systolic hypertension.

## Appendix

Multimedia Appendix 1 Assessment of bilateral blood pressure equality.

Multimedia Appendix 2 Normality analysis of acquired BP.

Multimedia Appendix 3 Correlational, actual indirect-direct error trend, paired simultaneous, and normalized % ratio difference comparative analysis of measured direct and indirect data BP.

Multimedia Appendix 4 Device initial algorithm vs intra-arterial pressure measures.

Multimedia Appendix 5 Device initial and central algorithms vs auscultatory pressure measures.

## Abbreviations

AAMI: Association for the Advancement of Medical Instrumentation
CHEP: Canadian Hypertension Education Program
ESH: European Society of Hypertension
FDA: Food and Drug Administration
SPRINT: Systolic Blood Pressure Intervention Trial
