# Supplementary figures and images for: A Comparison and Calibration of a Wrist-Worn Blood Pressure Monitor for Patient Management: Assessing the Reliability of Innovative Blood Pressure Devices
Source: J Med Internet Res. 2018 Apr 25;20(4):e111. doi: 10.2196/jmir.8009 (PMC5943631; doi:10.2196/jmir.8009)

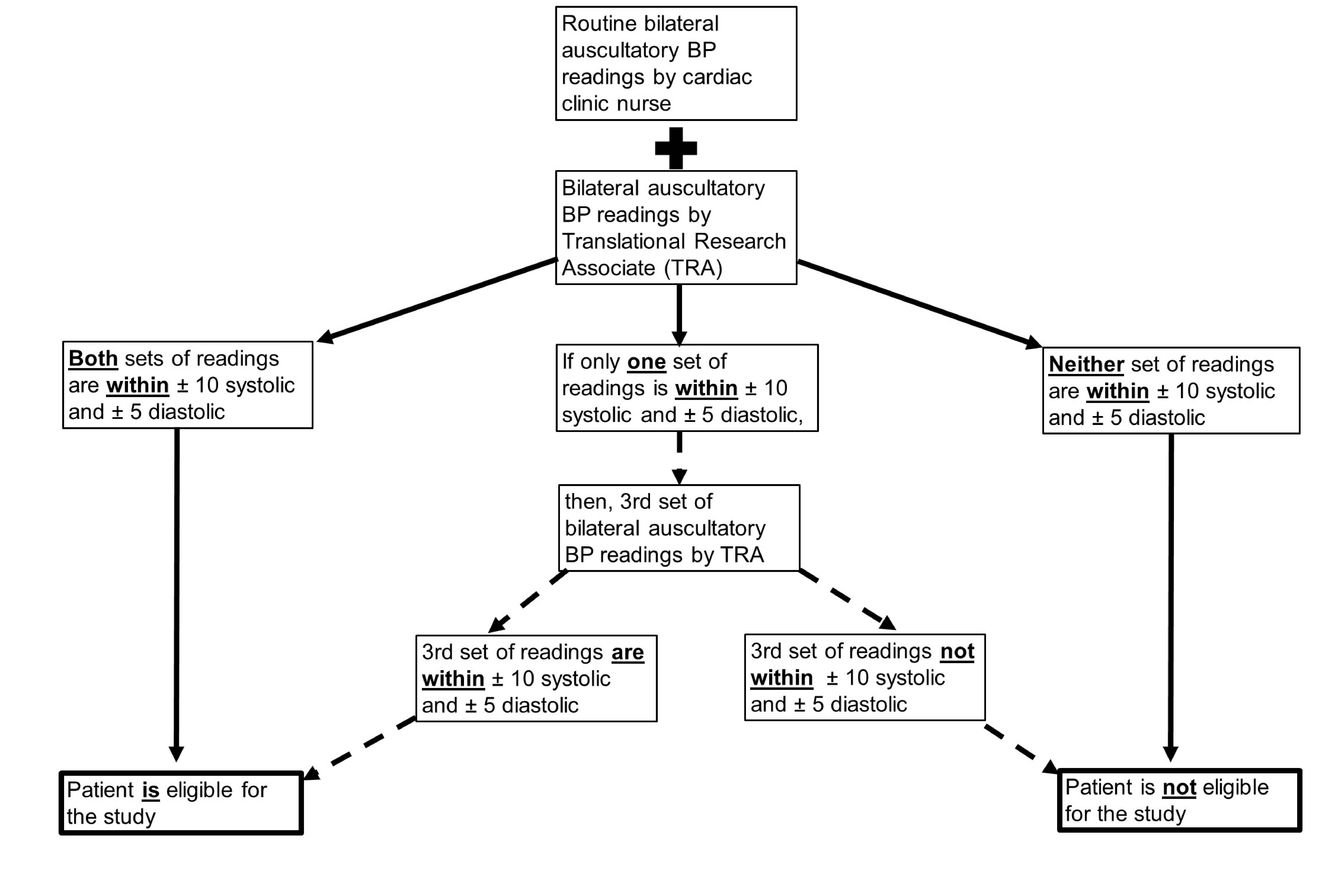

Supplement: Multimedia Appendix 1 [file jmir_v20i4e111_app1.jpg]

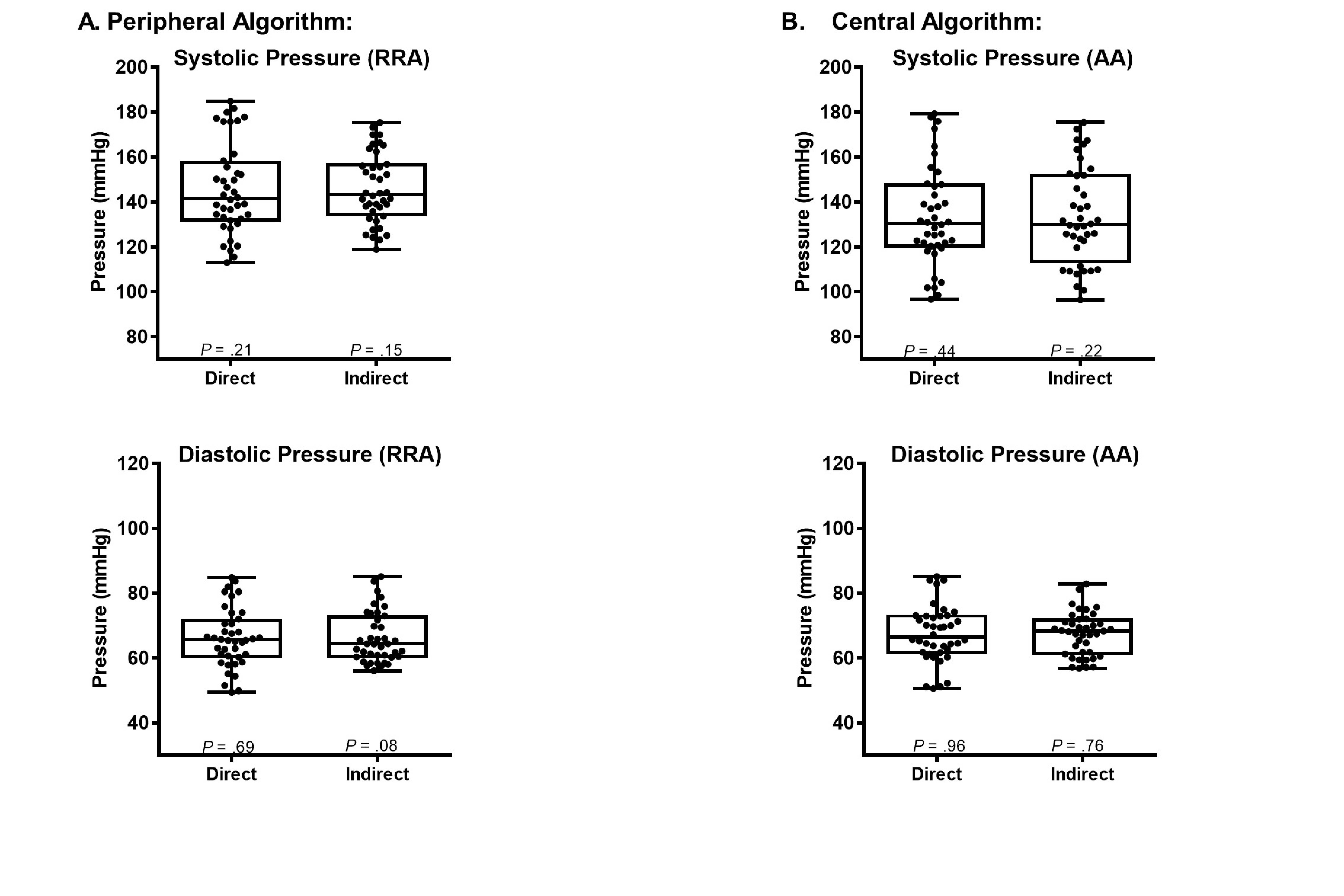

Supplement: Multimedia Appendix 2 [file jmir_v20i4e111_app2.jpg]

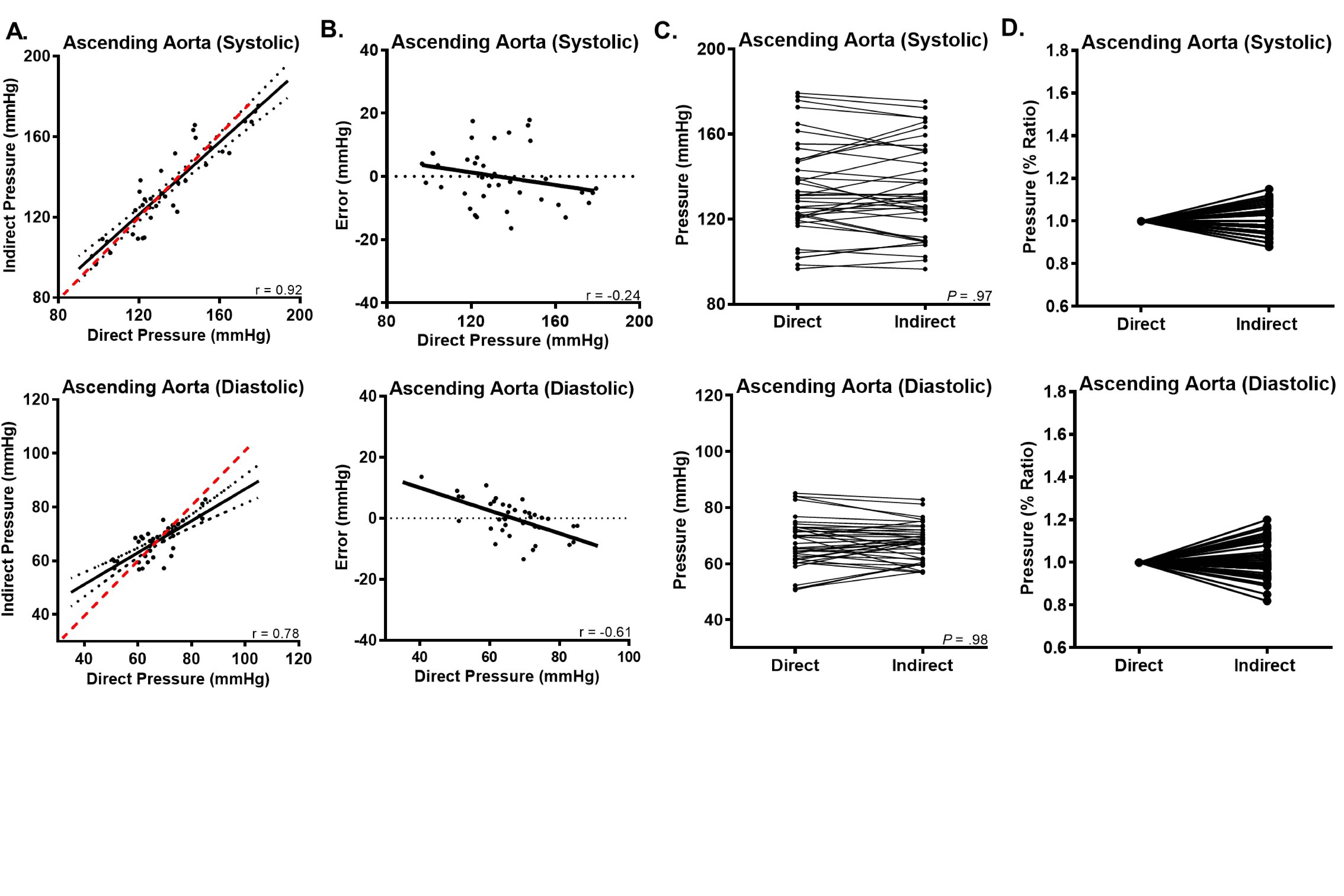

Supplement: Multimedia Appendix 3 [file jmir_v20i4e111_app3.jpg]

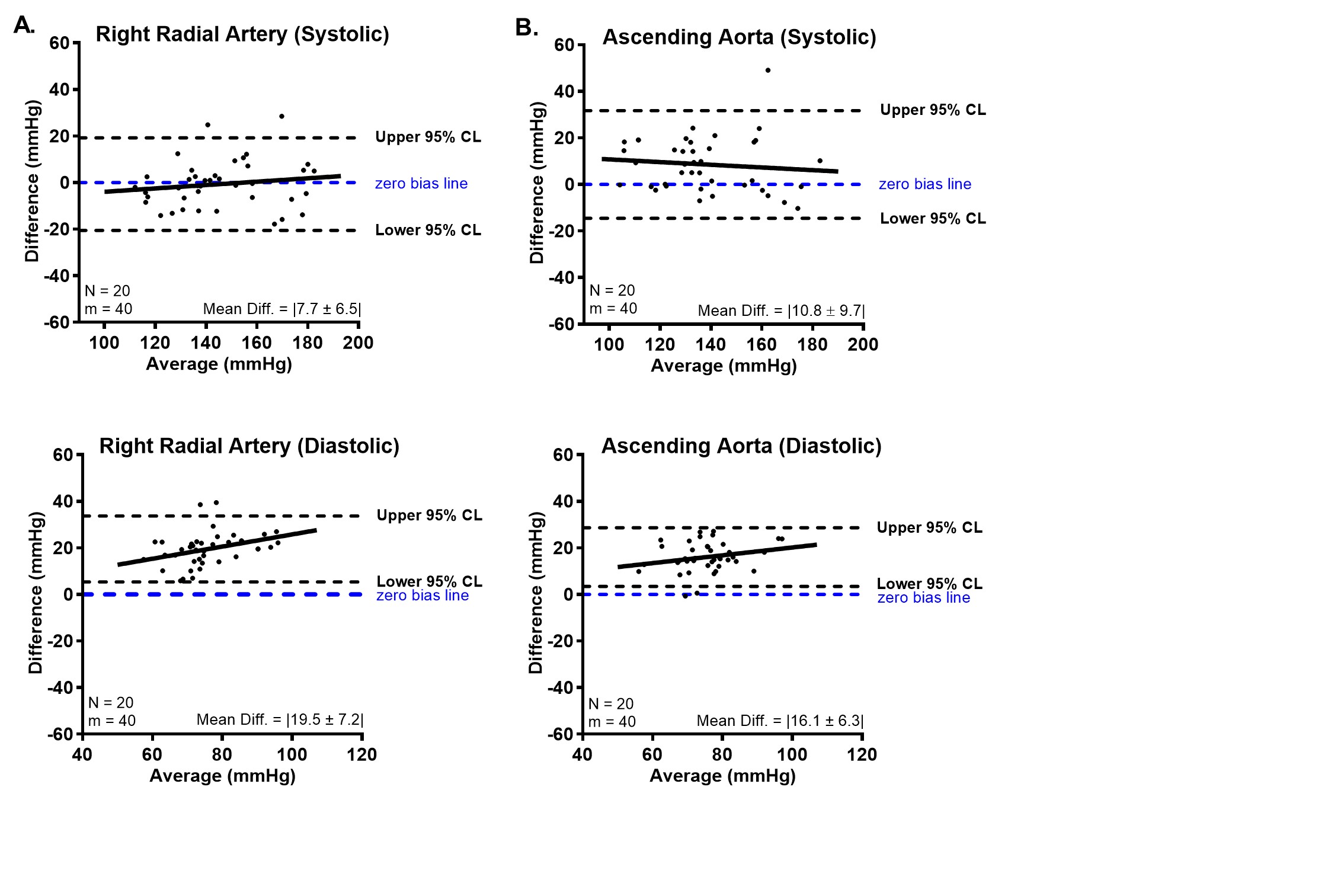

Supplement: Multimedia Appendix 4 [file jmir_v20i4e111_app4.jpg]

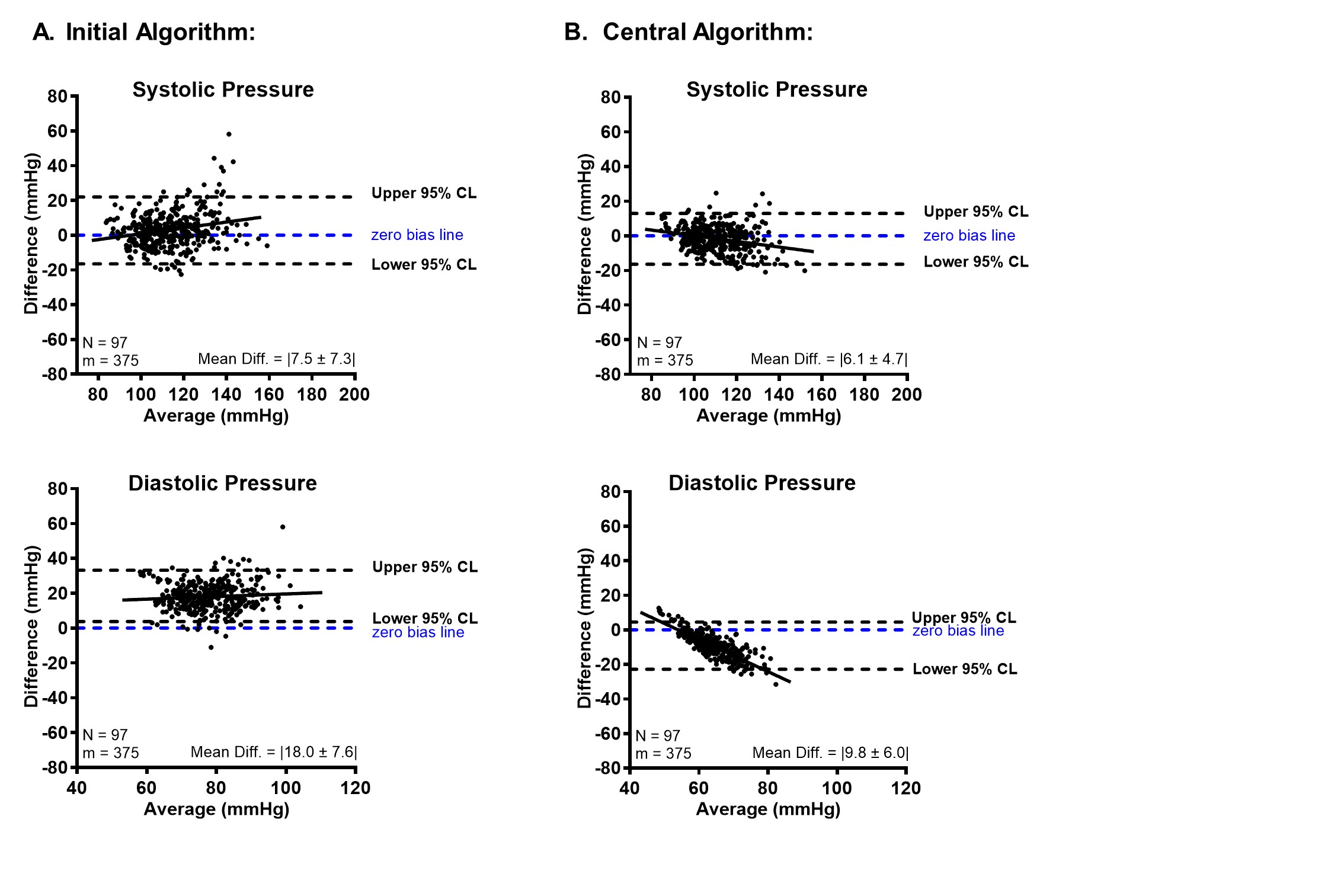

Supplement: Multimedia Appendix 5 [file jmir_v20i4e111_app5.jpg]
